# Molecular characterization of gut microbial structure and diversity associated with colorectal cancer patients in Egypt

**DOI:** 10.11604/pamj.2022.43.119.30037

**Published:** 2022-11-02

**Authors:** Mohamed Mohamed Adel El-Sokkary

**Affiliations:** 1 Department of Microbiology and Immunology, Faculty of Pharmacy, Mansoura University, Mansoura, Egypt

**Keywords:** Microbiota, colorectal cancer, Egypt

## Abstract

**Introduction:**

a large number of microbes colonizing the gut are highly diverse and complex in their structure, as this complex structure of gut microbiota acts as an indicator of a diseased state. Recently, there is a need for improved biomarkers for colorectal cancer (CRC) and advanced adenoma. Among the CRC associated organisms, bacteria are the most common causes of serious disease and deaths. To understand the dynamic interaction among bacteria colonizing the gut, different approaches have been implicated.

**Methods:**

in this study, faecal microbial markers were evaluated for detecting CRC. As most of these organisms are anaerobic, different molecular tools are of great values for rapid detection of these bacteria. Samples from Tumor Hospital were screened for the presence of different pathogens by both usual polymerase chain reaction (PCR) and a real-time assay.

**Results:**

in a total of 34 samples, by PCR method, bifidobacterium, fusobacterium and Escherichia coli (E. coli) were mainly identified in almost all samples. However, a clear variation in bacterial composition could be observed in Porphyromonas gingivalis, Prevotella intermedia and Peptostreptococcus magnus, where positive results could be detected only in diseased samples. In addition, E. faecium and E. saphenum were mainly identified in diseased samples. In contrast, providencia could be detected mainly in control samples. In realtime assay, the relative abundance was higher for fusobacterium and bifidobacterium markers in CRC patients compared to control samples. However, such increased in abundance has never been observed in both fusobacterium and bifidobacterium in the same sample.

**Conclusion:**

these results demonstrated increased abundance of fusobacterium or bifidobacterium can be considered as a sign for impairment or a diseased condition and the possibility of use of the faecal microbiotain CRC patients as a marker for detecting the disease.

## Introduction

Colorectal cancer (CRC) is one of the most common cancer in men and women worldwide [1]. Colorectal cancer onset and progression are induced by different mechanisms. Multi-factorial diseases such as colon cancer are influenced by physiological and environmental, in addition to genotypic changes [2,3]. This variation may be attributed to several measurable and unmeasurable factors. Habitat and diet may also be included as influencing factors [4]. As previously identified, in healthy individuals, in relation to diseased, the composition of the bacteria colonizing this area in the human body is variable [5]. Such variation emerges in altered bacterial signature abundance and the types of bacteria detected, which in turn favors the growth of some bacteria compared to the others. These new environmental changes with altered microbial composition is usually associated with metabolic activities resulting in the onset of inflammation, dysplasia, and cancer, which in turn can be used as a non-invasive detection marker in colorectal cancer patients. Stool population-based screening test for CRC has appeared as a test with a moderate sensitivity compared to the commercial faecal immunochemical test (FIT) [6].

Intestinal microorganisms can be involved with the production of toxic metabolites and the induction of a chronic inflammation state. In addition, a large number of microbes colonizing the gut are highly diverse and complex in their structure. This complex structure of gut microbiota is considered as an indicator of a diseased state. *Fusobacterium species* are among various organisms [7,8], which have been reported, in association with human colon cancer as potentiating factor for intestinal tumorigenesis. In addition, several bacteria in the faecal microbiota act as improved biomarker for the detection of both advanced adenoma and colorectal cancer (CRC) [9-11]. To understand the dynamic interaction among bacteria colonizing the gut, different approaches have been implicated. For identifying novel tumor-associated microbes, in a culture-independent approach, most recently, metagenomic analysis has become a sensitive method producing a disease characteristic sequence signature. Several previous reports have indicated the possible application of microbiota profiling as a tool for detection of CRCs and its applicability in faecal samples [11-13]. Despite these advances, for early diagnosis, additional studies in humans and animal models are needed to investigate the relationship between CRC and the gut microbiota, which enhance the development of alternate therapies based on the results obtained in these studies. For these reasons, a highly sensitive non-invasive accurate test for both advanced adenoma and CRC and is highly desirable. These methods with their potential biomarkers developed for key bacterial that play a potential role in CRC development will be of a great importance.

The aim of our study is to apply these new methodologies to detect different potential biomarkers, which are associated with CRC development in samples isolated from an Egyptian hospital.

## Methods

**Specimen collection, processing and genomic deoxyribonucleic acid (DNA) extraction:** twenty-seven different stool samples were taken form CRC patients, in addition to 7 control stool samples. After stool collection, samples were delivered from the hospital within 12 hours and stored at -80°C immediately in our lab until further analysis. Genomic DNAs were extracted from stool samples using ZR Faecal DNA MiniPrep Kit (Zymo Research, USA), according to manufacturer´s instructions. Purified DNAs were frozen at -80°C in 40 μl aliquots for the following PCR. The concentration of gDNA was determined a using Nanodrop (OPTIZEN NanoQ, Mecasys).

This study was performed under the ethical guidelines adopted by “The Research Ethics Committee, Faculty of Pharmacy, Mansoura University” which is in accordance with the Code of Ethics of World Medical Association (Declaration of Helsinki involving use and handling of human subjects).

**Polymerase chain reaction (PCR) amplification of strain specific genes:** amplification of genomic DNA was performed using primers listed in Table 1. The reaction mixture was prepared starting from gDNA as a template, in a reaction mixture containing 0.5 μM of each primer, 1.5 mM MgCl_2_, 0.2 mM dNTPs, 1 U Taq polymerase (Thermo scientific Dream Taq Green DNA polymerase), 2 μl of template DNA and nuclease free water for a total volume of 25 μl per reaction. Each PCR was performed using Cycler 003 PCR Machine (A & E Lab (UK)). PCR reactions began with 5 minutes of primary denaturation at 94°C followed by 35 cycles of 94°C for 30s, annealing temp (Table 1) for 30 s and 72°C for 30 s and a final extension at 72°C for 10 min.

**Table 1:** different primers used in this work to detect different species of bacteria

Primer name	Primer sequence		Annealing Tm	Size bp
*ddl E. faecalis*	ddl E F	ATCAAGTACAGTTAGTCTT	44	940
	ddl E R	ACGATTCAAAGCTAACTG		
*Ddl E. faecium*	ddl E F	GCAAGGCTTCTTAGAGA	46.5	564
	ddl E R	CATCGTGTAAGCTAACTTC		
*E. coli*	TEcol553	TGGGAGCGAAAATCCTG	47.5	219
	TEcol754	CAGTACAGGTAGACTTCTG		
*Bifidobacterium*	g-Bifid-F	CTCCTGGAAACGGGTGG	51	551
	g-Bifid-R	GGTGTTCTTCCCGATATCTACA		
*Porphyromonas gingivalis*	Pg593-1	AATCGTAACGGGCGACACAC	53	594
	Pg593-2	GGGTTGCTCCTTCATCACAC		
*M. timidum*	tim44F	AAGCTTGGAAATGACGC	46	524
	tim568R	CCTTGCGCTTAGGTAA		
All bacteria	F	GAGTTTGATCCTGGCTCAG	51	312
	R	GCTGCCTCCCGTAGGAGT		
*Prevotella intermedia*	Pin-F1	CGAACCGTCAAGCATAGGC	54	368
	Pin-R1	AACAGCCGCTTTTAGAACACAA		
*Peptostreptococcus magnus*	Pmag-1	CGGGNTTTAGTAGACAGAAG	50	565
	Pmag-2	CAGTTTCCAATGCTTTACGG		
*Fusobacterium*	F	GGATTTATTGGGCGTAAAGC	51.5	162
	R	GGCATTCCTACAAATATCTACGAA		
*E. saphenum*	sap156F	AACCACATAAAATCATAGG	43	828
	sap964R	ATACCCGATTAAGGGTAC		
*Providencia*	sp16s-F1	ACCGCATAATCTCTTAGG	43.5	514
	Psp16s-R2	CTACACATGGAATTCTAC		

**Real time PCR test:** the relative abundance of *fusobacterium, bifidobacterium*, and *E. coli* compared to 16S housekeeping gene was determined using quantitative PCR (Qpcr) technique. In 25μl reaction volume, 40-80 ng of extracted faecal DNA were added to 12.5μl (2x) SYBR Green PCR master mix (Fermentas Co.), 1.5μl of each forward and reverse primer (10μmol each) and 7.5μl nuclease free H_2_O. The realtime experiments were set up on MyGo real time PCR machine using optical tube and cap strips under the following reaction conditions: 95°C for 1 min, followed by 45 cycles of 95°C for 20 s, annealing temperature °C for 20 s, 72°C for 40 s. Ct value is the number of cycles at which the fluorescent signal exceeds the threshold cycle. For detection of amplification specificity, melting curves were observed. Microbial markers abundance was calculated using the 2^-ΔCt^ method (where ΔCt=the average Ct value of each target- the average Ct value of total bacteria). Amplification, detection and analysis of DNA was performed for the real-time PCR results using the MyGo real time PCR machine software.

## Results

**Polymerase chain reaction (PCR) as a primary test:** by usual uniplex PCR, a total of 27 stool samples, taken from patients admitted to the Mansoura Tumor Hospital, in addition to 7 control samples were subjected to PCR testing of 11 different bacterial species, associated with CRC disease (Table 2). These bacteria include: *E. faecalis, E. faecium, Fusobacterium* sp., *E. saphenum, Bifidobacterium* sp., *E. coli, Porphyromonas gingivalis, M. timidum, Eubacteria, Prevotella intermedia, Providencia* sp. and *Peptostreptococcus magnus*. In a total of 34 samples, *Fusobacterium* sp., *Bifidobacterium* sp. and *E. coli* were mainly identified in 31, 33 and 34 samples respectively. Lower prevalence was observed in *Porphyromonas gingivalis, Prevotella intermedia*, and *Peptostreptococcus magnus*, where positive results could be detected only in diseased samples in 10, 1, and 7 samples, respectively. *Peptostreptococcus magnus* and *Prevotella intermedia* could be detected in only female diseased samples, 7 and 1, respectively. *Porphyromonas gingivalis, E. saphenum* and *E. faecium* could be detected in 4, 1 and 5 male, in addition to 6, 6 and 3 female samples respectively. Control samples were positive for *M. timidum, E. saphenum* and *E. faecium* in, 2, 2 and 2 samples respectively. Interestingly, a higher abundance of *Providencia* sp. could be detected mainly in control samples (85.7%) compromising 6 isolates, while 5 were identified in diseased ones (18.5%).

**Table 2:** different species prevalence in diseased samples compared to control

Organism	Gender of diseased				Control 7	%
	**Male 12**	**%**	**Female 15**	**%**		
*E. faecalis*	0	0	1	6.6	0	0
*E. faecium*	5	42	3	20	2	28.6
*E. coli*	12	100	15	100	7	100
*Bifidobacterium*	11	92	15	100	7	100
*Porphyromonas gingivalis*	4	33	6	40	0	0
*M. timidum*	3	25	3	20	2	28.6
*Eubacteria*	12	100	15	100	7	100
*Prevotella intermedia*	0	0	1	6.6	0	0
*Peptostreptococcus magnus*	0	0	7	46.6	0	0
*Fusobacterium*	10	83	14	93.3	7	100
*E. saphenum*	1	8	6	40	2	28.6
*Providencia*	2	16	3	20	6	85.7

In this study, *E. coli* could be identified in all isolates. In addition, *Bifidobacterium* sp. could not be detected in only one male sample. However, *Fusobacterium* sp. was not detected in two males and one female sample. For this reason, as a sharp variation in the detected samples could not be resolved, realtime technique was implemented to find out such variation.

**Figure 1:**
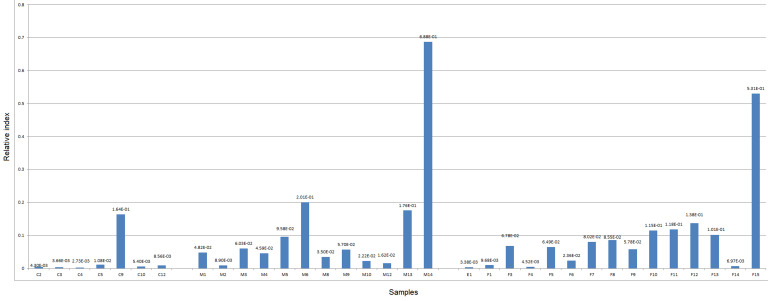
relative abundance of *E. coli* in male and female diseased compared to control

**Figure 2:**
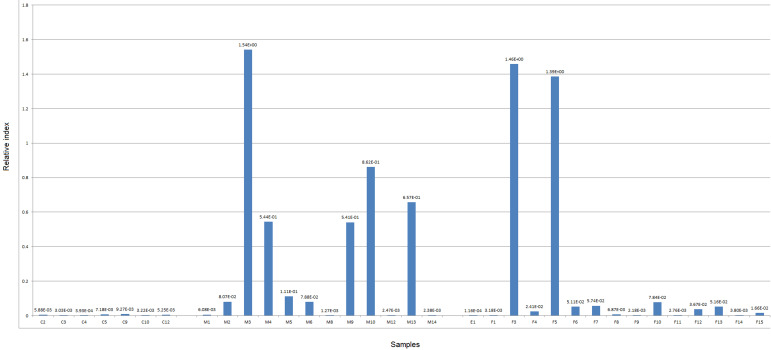
relative abundance of *Bifidobacterium* sp. in male and female diseased compared to control

**Figure 3:**
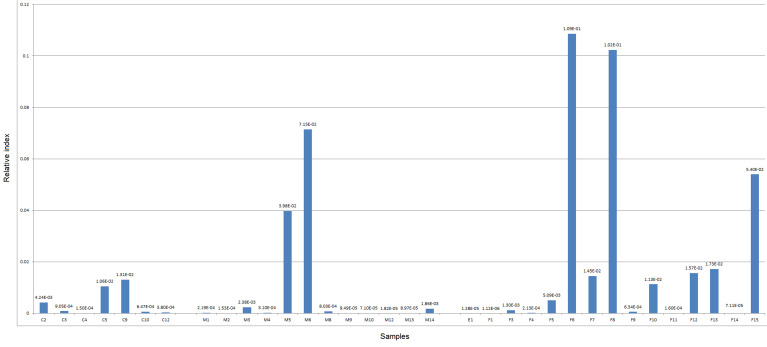
relative abundance of *Fusobacterium* sp. in male and female diseased compared to control

**Realtime PCR test:** the relative abundance of four microbial markers for fusobacterium, bifidobacterium and *E. coli* were determined in 34 individuals, including 27 patients with CRC and 7 healthy controls. In some of the disease samples, realtime PCR results demonstrated a higher abundance of *Fusobacterium* sp., compared to control samples. Similarly, *Bifidobacterium* sp. was higher in the rest of diseased samples. Interestingly, *E. coli* abundance was higher in most of diseased samples compared to control (Figure 1, Figure 2, Figure 3).

## Discussion

Recently developed molecular methods, such as next generation sequencing techniques were implicated in several studies [5,14]. The aim of these studies was to investigate the correlation between clinical cases of CRC, dysbiosis and dysbiosis and gut microbiota. As previously reported, elevated abundances in patients with CRC were identified [5], including the widely reported *F. nucleatum, Porphyromonas asaccharolytica, P. micra, Desulfovibrio desulfuricans* and *Akkermansia muciniphila*. In addition, diversity analysis showed tumour microbiota, enriched significantly with *bifidobacteriaceae*, enterococcus, proteus and escherichia/shigella [14]. However, in another study, the majority of enriched microbes were classified at the family level as *fusobacteriaceae, lachnospiraceae, peptoniphilaceae, porphyromonadaceae, bacteroi- daceae, prevotellaceae, peptostreptococcaceae, ruminococcaceae, Bacillales incertaesedis and streptococcaceae*. In contrast, among the group of decreased microbes, most were classified into *bifidobacteriaceae, lachnospiraceae, bacteroidaceae, ruminococcaceae, eubacteriaceae* and *streptococcaceae* [15].

The colon has a reductive environment devoid of oxygen. Thus, most microbial populations are strictly anaerobic. In this study, the composition of the human intestinal microbiota, detected in CRC patients to healthy subjects was compared adapting the culture-independent PCR and RT-qPCR methods. As a result, in a total of 34 samples, *Fusobacterium* sp., *Bifidobacterium* sp. and *E. coli* were mainly identified in 31, 33 and 34 samples respectively. However, these results obtained from PCR could not indicate a significant variation in samples of diseased and healthy. Therefore, a real time PCR test was needed to detect this variation.

Acetate, propionate, and butyrate are principally produced by short chain fatty acid microorganism producers [16,17]. These bacteria are associated with CRC main symptoms including bleeding and colorectal tissue rupture, resulting in a newly modified microenvironment, which in turn induces a selective pressure on the components of the gut microbiota enhancing the growth of some bacteria such as *F. nucleatum* replacing the typical commensal intestinal flora [18]. Therefore, the prevalence of *F. nucleatum* in CRC may be related to its invasive and inflammatory properties. In addition, it has been previously observed, in colorectal carcinoma (CRC), the overabundance *Fusobacterium nucleatum* in tissue compared to adjacent non-tumor gut mucosal control tissue from the same subjects [8]. This over-representation of *Fusobacterium* sp. in CRC tumors has also been previously documented in many studies [11,13,19,20], which is usually associated with a pro-inflammatory expression signature. Furthermore, as previously reported, a very important role is carried out by some pathogens, mainly *Fusobacterium nucleatum* in the development of CRC. In this study, this observation was verified in CRC and control subjects using a quantitative PCR assay, targeting *Fusobacterium* sp. By qPCR assay, a significantly higher percentage of *Fusobacterium* sp. could be traced in the fecal samples of CRC patients, compared to control samples.

In this study, *Bifidobacterium* sp. could be quantified in a higher amount in the fecal samples of CRC patients, compared to control samples. This variation may be attributed to the influence produced by the environment created due to CRC significantly increasing their numbers in some samples. The daily renewal of the colon epithelial cells may be activated due to the environment created based on CRC inducing the overabundance of lactic acid-producing bacteria, which may be affected, thus allowing their growth in higher numbers. *Bifidobacterium* sp., as a member of lactic acid-producing bacteria has been suggested to strengthen and maintain the mucosal barrier function and the daily renewal of the colon epithelial cells. This may be achieved through the production of mucin, antimicrobial peptides, and tight-junction proteins. In addition, the growth of *Bifidobacterium* sp. stimulates the generation of nicotinamide adenine dinucleotide phosphate (NADPH)-dependent reactive oxygen species and intestinal stem cell proliferation [11,21].

Concerning other microbial biomarkers, in this study, by performing PCR, in a total of 34 samples, *Porphyromonas gingivalis, Prevotella intermedia*, and *Peptostreptococcus magnus*, could be detected only in diseased samples. In addition, a higher prevalence compared to control was observed in *Porphyromonas gingivalis, E. saphenum* and *E. faecium*. In contrast, providencia, a species usually observed in normal gut microbiota, could be detected mostly in control samples, where 6 positive samples observed. These results are in accordance with some previous studies, which reflect the alteration in the mucosa-adherent microbiota of CRC patients exhibiting an increased number of several bacteria with putative carcinogenic role such as *Porphyromonas* sp., *Fusobacterium* sp., *Peptostreptococcus* sp. and *Mogibacterium* sp., while *Faecalibacterium* sp. and *Blautia* sp., appeared diminished [22-25].

Numerous tests for early CRC detection, employing such non-invasive biomarkers have been proposed and clinically studied. As previously documented, only 73.8% detection sensitivity could be obtained using current fecal immunochemical (FIT) testing for CRC (100ng/mL), in comparison to 92.3% in case of stool-based DNA assay screening bone morphogenetic protein 3 (BMP3), KRAS, aberrant NDRG4 and methylation [26], however with a limited diagnostic screening for early disease detection. In contrast, miRNAs, if combined with other microRNAs or other forms of biomarker for diagnostic and predictive purposes may show greater specificity and sensitivity. The significantly increased IL8, MMP2 and BAFF, and decreased APRIL expression were tumor-specific with no statistically significant differences [27] have been found across the staging groups. Other candidate gene PVT1, an oncogenic lncRNA, was used as a biomarker for prediction, diagnosis and prognosis [28]. Some of these studies generated promising early results, however, very few of the proposed tests have been transformed into clinically validated diagnostic/screening techniques. Despite being expensive and technically complex, DNA-based tests, multitarget stool test or blood test for methylated septin 9 showed a good diagnostic performance, as indicated by food and drug administration. However, recently, the protein (haemoglobin) detection-based faecal immunochemical test (FIT) represents the most cost-effective option for non-invasive CRC screening, In addition to the confirmatory invasive colonoscopy [29].

**Limitation of microbial detection methods:** numerous studies have reported associations between microbial markers, such as *F. nucleatum*, or *E. coli* and CRC, however, more efforts are needed to find out a universal microbial marker for CRC detection. Several limitations associated with the variability of the microbiota composition should be taken into consideration. In addition, some other factors, related to the individual variations such as age, sex, diet, genetic, medication use, lifestyle or geographical location represent also additional challenges [30].

**Disclosure:** this work was performed at Microbiology Department, Faculty of Pharmacy, Mansoura University, Egypt.

## Conclusion

The prevalence of fusobacterium in fecal microbiota, in addition to some other species such as *Porphyromonas gingivalis, Prevotella intermedia, Peptostreptococcus magnus, Porphyromonas gingivalis, E. saphenum* and *E. faecium* could be used as a possible fecal marker for the pre-diagnosis of CRC.

### What is known about this topic

- A large number of microbes colonizing the gut are highly diverse and complex in their structure;
- This complex structure of gut microbiota acts as an indicator of a diseased state;
- Microbial diversity screening systems are essential to ensure quality in the diagnosis and treatment of non-communicable diseases.

### What this study adds

- To improve the control and management of patients with chronic non-communicable diseases, new methods has to be tested for their validity as diagnostic tools;
- Molecular screening of gut microbiota represents a new methodology, described previously in not so many studies on samples, isolated from Egyptian hospitals;
- The prevalence of fusobacterium in fecal microbiota, in addition to some other species could be used as a possible fecal marker for the pre-diagnosis of CRC.
